# Stem cell senescence. Effects of REAC technology on telomerase-independent and telomerase-dependent pathways

**DOI:** 10.1038/srep06373

**Published:** 2014-09-16

**Authors:** S. Rinaldi, M. Maioli, G. Pigliaru, A. Castagna, S. Santaniello, V. Basoli, V. Fontani, C. Ventura

**Affiliations:** 1 Department of Regenerative Medicine, Rinaldi Fontani Institute, Viale Belfiore 43, 50144 Florence, Italy; 2 Department of Anti Aging Medicine, Rinaldi Fontani Institute, Viale Belfiore 43, 50144 Florence, Italy; 3 Research Department, Rinaldi Fontani Foundation NPO, Viale Belfiore 43, 50144 Florence, Italy; 4 Department of Biomedical Sciences, University of Sassari, Viale San Pietro 43/B, 07100 Sassari, Italy; 5 Stem Wave Institute for Tissue Healing (SWITH), Gruppo Villa Maria and Ettore Sansavini Health Science Foundation NPO, via Provinciale per Cotignola 9, 48022 Lugo (Ravenna), Italy; 6 National Institute of Biostructures and Biosystems at the Department of Experimental, Diagnostic and Specialty Medicine, S. Orsola - Malpighi Hospital, University of Bologna, Via Massarenti 9, 40138 Bologna, Italy; 7 These authors contributed equally to this work.

## Abstract

Decline in the gene expression of senescence repressor Bmi1, and telomerase, together with telomere shortening, underlay senescence of stem cells cultured for multiple passages. Here, we investigated whether the impairment of senescence preventing mechanisms can be efficiently counteracted by exposure of human adipose-derived stem cells to radio electric asymmetrically conveyed fields by an innovative technology, named Radio Electric Asymmetric Conveyer (REAC). Due to REAC exposure, the number of stem cells positively stained for senescence associated β-galactosidase was significantly reduced along multiple culturing passages. After a 90-day culture, REAC-treated cells exhibited significantly higher transcription of Bmi1 and enhanced expression of other stem cell pluripotency genes and related proteins, compared to unexposed cells. Transcription of the catalytic telomerase subunit (TERT) was also increased in REAC-treated cells at all passages. Moreover, while telomere shortening occurred at early passages in both REAC-treated and untreated cells, a significant rescue of telomere length could be observed at late passages only in REAC-exposed cells. Thus, REAC-asymmetrically conveyed radio electric fields acted on a gene and protein expression program of both telomerase-independent and telomerase-dependent patterning to optimize stem cell ability to cope with senescence progression.

In human adult tissues a small population of resident mesenchymal stem cells (hMSCs) plays an important role in replacing old or damaged cells to maintain tissue integrity, and combat age-related processes^1^,^2^,^3^,^4^,^5^,^6^. The regenerative capability of hMSCs is not only related to the tissue source (adipose tissue, bone marrow, placental foetal membranes, amniotic fluid), but also to the age of tissue donors^7^, since even stem cells undergo cellular senescence, which deeply affects their own self-renewal and differentiation potential^7^,^8^. Moreover, in most clinical trials hMSCs are subjected to long-term expansion *ex vivo*, in order to yield a large number of viable elements or to precondtion stem cells with various strategies (i.e. chemical or physical agents) optimizing their commitment and/or paracrine release prior to transplantation. To this end, stem cell culturing for multiple passages is both a risk of, and a well-established model for cell senescence *in vitro*^9^,^10^. Adipose-derived hMSCs (ADhMSCs) are increasingly used as a source for cell therapy, due to the ease of tissue harvesting and their robust multipotency. Nevertheless ADhMSCs also undergo significant senescence after multiple passages in culture^9^,^11^,^12^. This observation suggests that caution should be exercised when using ADhMSCs after long passage times and prompts the needs for novel strategies counteracting senescence during the expansion of such a promising source of cell therapy.

Molecular control of stem cell fate and senescence is mainly regulated by two different, telomerase-independent and telomerase-dependent, pathways. In the telomerase-independent senescence pathway, epigenetic events, such as histone modification, have been implicated. It follows that genes influencing chromatin remodeling and gene expression regulation may be directly involved in decisions that affect stem cell fate, including self-renewal and senescence^13^,^14^,^15^. Within this context, Bmi1, a member of the *Polycomb* and *Trithorax* families group of repressors, has been recently shown to be an essential factor for the self-renewal of adult murine hematopoietic stem cells, and neuronal stem cells, acting as a repressor of senescence^16^,^17^,^18^,^19^. The pluripotency transcription factors Nanog, Oct4, Sox2 and cMyc, together with polycomb repressive complexes, have also been found to regulate stem cell pluripotentiality and differentiation^16^,^17^,^20^,^21^.

Telomerase is a specialized ribonucleoprotein composed of telomerase reverse transcriptase (TERT), an intrinsic RNA template (TR), and several associated proteins^22^. Its main function is to stabilize telomeres, which shorten with each round of cell division^23^, thus protecting chromosomes from recombination and end-to-end fusion. Telomerase is expressed in highly proliferating cells throughout the developing embryo, while it is dramatically down-regulated as cells differentiate, being undetectable in many adult somatic cells^24^,^25^,^26^. Otherwise, studies on cancer cells and engineered somatic cells overexpressing telomerase have shown that this enzyme can confer an immortal phenotype^27^,^28^. Therefore, developing a tool that can revert the molecular mechanisms underlying stem cell senescence *in vitro* may pave the way to affordable strategies of stem cell expansion, improving the outcome of cell therapy efforts.

In previous work, we have shown that Radio Electric Asymmetric Conveyer (REAC), an innovative technology^29^,^30^ delivering radio electric asymmetrically conveyed fields with its conveyer electrodes immersed into the culture medium^31^, remarkably enhanced the commitment of mouse embryonic stem cells toward different lineages, including cardiogenic, neurogenic and skeletal myogenic, without the aid of chemical compounds or viral vector-mediated gene delivery^31^. Moreover, exposure to REAC afforded a direct reprogramming of human dermal skin fibroblasts^32^ ensuing into a rapid, high-throughput and stable commitment towards cardiogenesis, skeletal myogenesis, and neurogenesis^32^. REAC protocols of regenerative medicine have also shown a clear efficacy in abating the effects of the aging process *in vitro*^33^,^34^,^35^.

In the present work, we investigated whether the REAC technology may be exploited to counteract senescence in ADhMSCs during prolonged expansion *in vitro*, acting on major modulators of stem cell aging and self-renewal, including the epigenetic regulator Bmi1, and TERT, as well as the telomere length.

## Results

To expose ADhMSCs to REAC, we used a specific REAC treatment named tissue optimization – regenerative protocol (TO-RGN). The REAC technology was originally designed to convey the radio electric currents resulting from the interaction between the weak electromagnetic field produced by the instrument, with a radiated power of about 2 mW, and the electromagnetic field generated by the human body, with a radiated power of about 54 mW^36^.

### REAC TO-RGN influences beta-galactosidase staining during prolonged cultures passages

**Figure 1:**
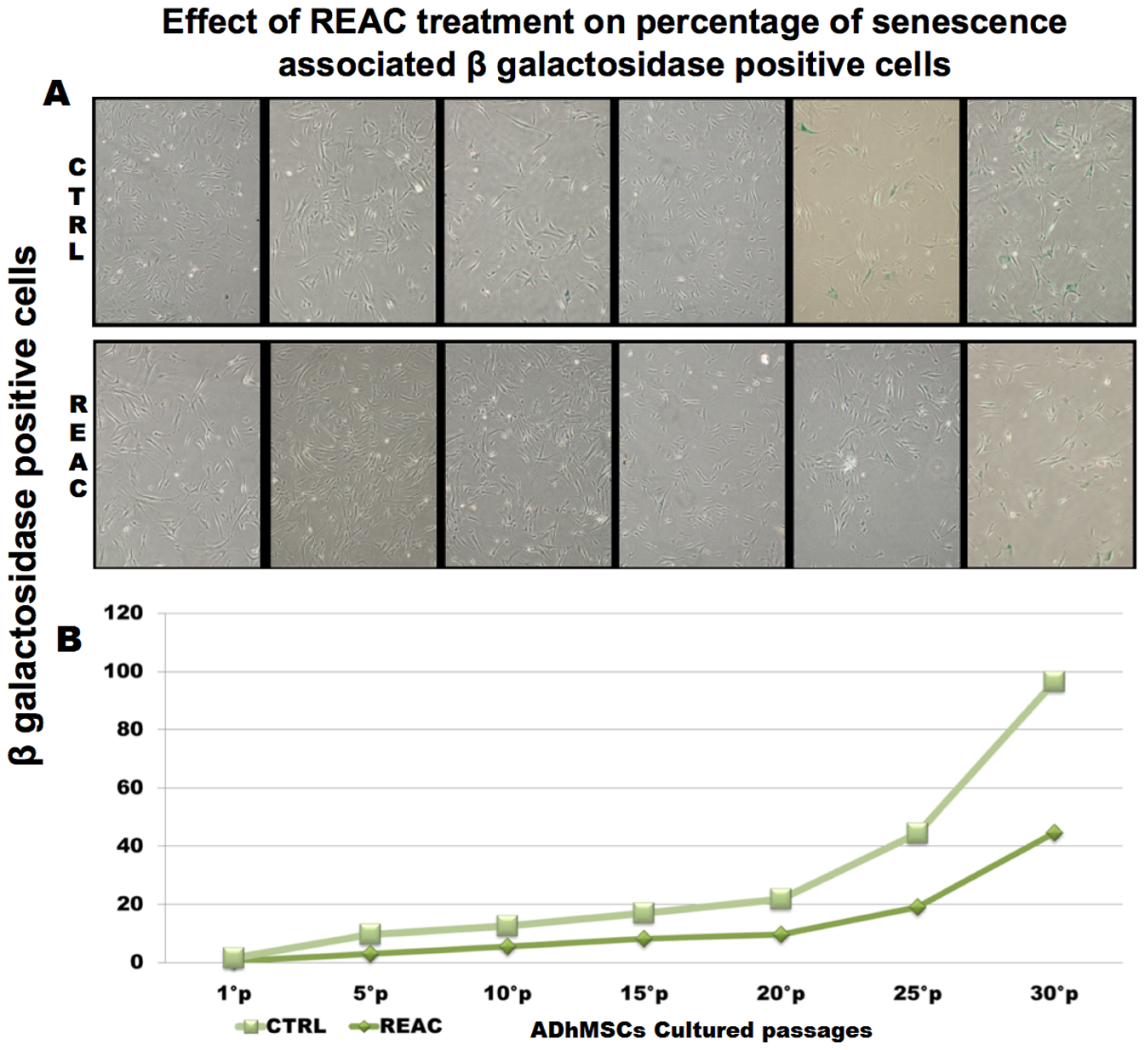
REAC TO-RGN exposure decreases SA-β-Gal staining along multiple culturing passages. ADhMSCs at passages 1, 5, 10, 15, 20, 25 and 30, reaching 80% confluence, were exposed for 12 hours in the absence or presence of REAC (panel A, representative of six separate experiments). The percentage of cells positively stained (blue color) for SA-β-Gal was assessed as described in the Methods section (panel B, mean ± S.E.; n = 6; P < 0.05).

ADhMSCs were subjected to a prolonged culture in the absence or presence of REAC TO-RGN, and the expression of senescence-associated β-galactosidase (SA-β-gal) was assessed at passages 1, 5, 10, 15, 20, 25 and 30 (Figure 1A). As revealed by the quantitative analysis over a culturing period of 50 days (Figure 1B), the number of SA-β-gal stained cells in REAC exposed ADhMSCs was significantly reduced along all the culturing passages, as compared to control untreated cells. In particular this differences between the two cell populations become more evident at late passages (25–30).

### REAC TO-RGN affects the expression of a transcriptional regulator involved in chromatin remodeling

**Figure 2:**
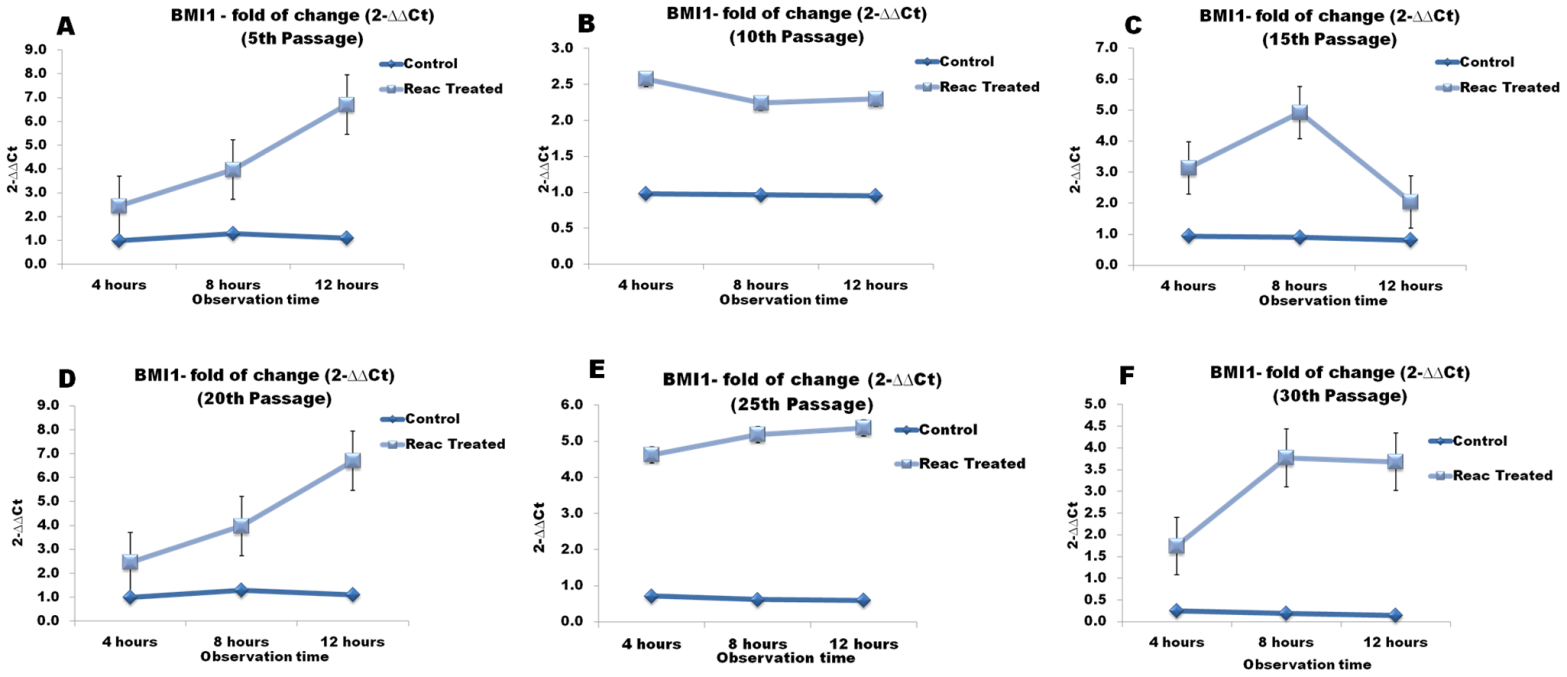
Effect of REAC TO-RGN exposure on the expression Bmi1. ADhMSCs at passages 5 (panel A), 10 (panel B), 15 (panel C), 20 (panel D), 25 (panel E) and 30 (panel F), reaching 80% confluence, were exposed for 4, 8 or 12 hours in the absence or presence of REAC. The amount of Bmi1 mRNA from control or REAC-treated cells was normalized to HPRT1, and was plotted as fold change relative to the mRNA expression at time 0, defined as 1. All the REAC-treated cells at each time point were significantly different from each control untreated cells (mean ± S.E.; n = 6) (P < 0.05).

Real-time PCR analysis was conducted to assess the gene expression of Bmi1 in both control cells and ADhMSCs that had been exposed to REAC TO-RGN for 4, 8 or 12 hours at passages 5, 10, 15, 20, 25 and 30. In unexposed cells, transcript levels progressively declined over culturing time (Figure 2, panel A–F). On the contrary, REAC TO-RGN treatment significantly counteracted the passage-dependent down-regulation of Bmi1 transcription, at all the investigated passages (Figure 1, panels A–F).

### REAC TO-RGN influences the expression of genes controlling stem cell pluripotency

**Figure 3:**
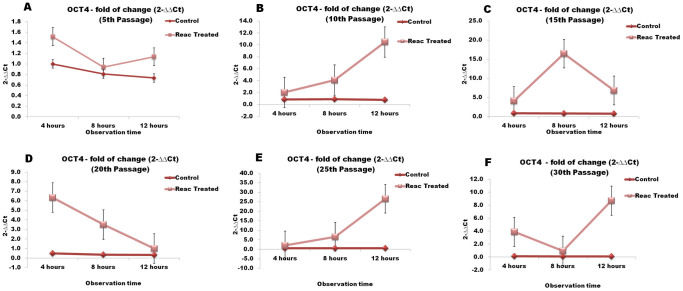
REAC TO-RGN treatment modulates the gene expression of Oct4. ADhMSCs at passages 5 (panel A), 10 (panel B), 15 (panel C), 20 (panel D), 25 (panel E) and 30 (panel F), reaching 80% confluence, were exposed for 4, 8 or 12 hours in the absence or presence of REAC. The amount of Oct4 mRNA from control or REAC-treated cells was normalized to HPRT1, and was plotted as fold change relative to the mRNA expression at time 0, defined as 1. All the REAC-treated cells at each time point were significantly different from each control untreated cells (mean ± S.E.; n = 6) (P < 0.05).

**Figure 4:**
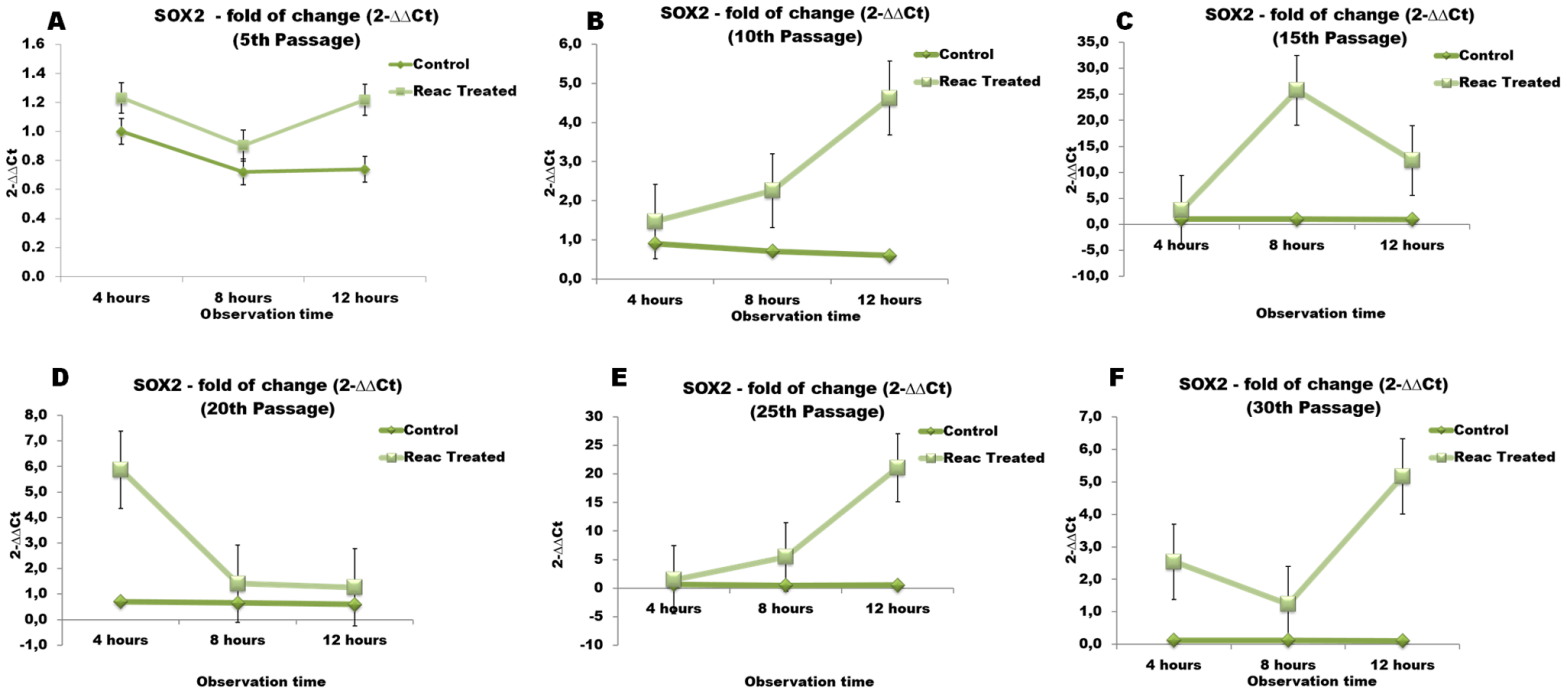
REAC TO-RGN treatment modulates the gene expression of Sox2. ADhMSCs at passages 5 (panel A), 10 (panel B), 15 (panel C), 20 (panel D), 25 (panel E) and 30 (panel F), reaching 80% confluence, were exposed for 4, 8 or 12 hours in the absence or presence of REAC. The amount of Sox2 mRNA from control or REAC-treated cells was normalized to HPRT1, and was plotted as fold change relative to the mRNA expression at time 0, defined as 1. All the REAC-treated cells at each time point were significantly different from each control untreated cells (mean ± S.E.; n = 6) (P < 0.05).

**Figure 5:**
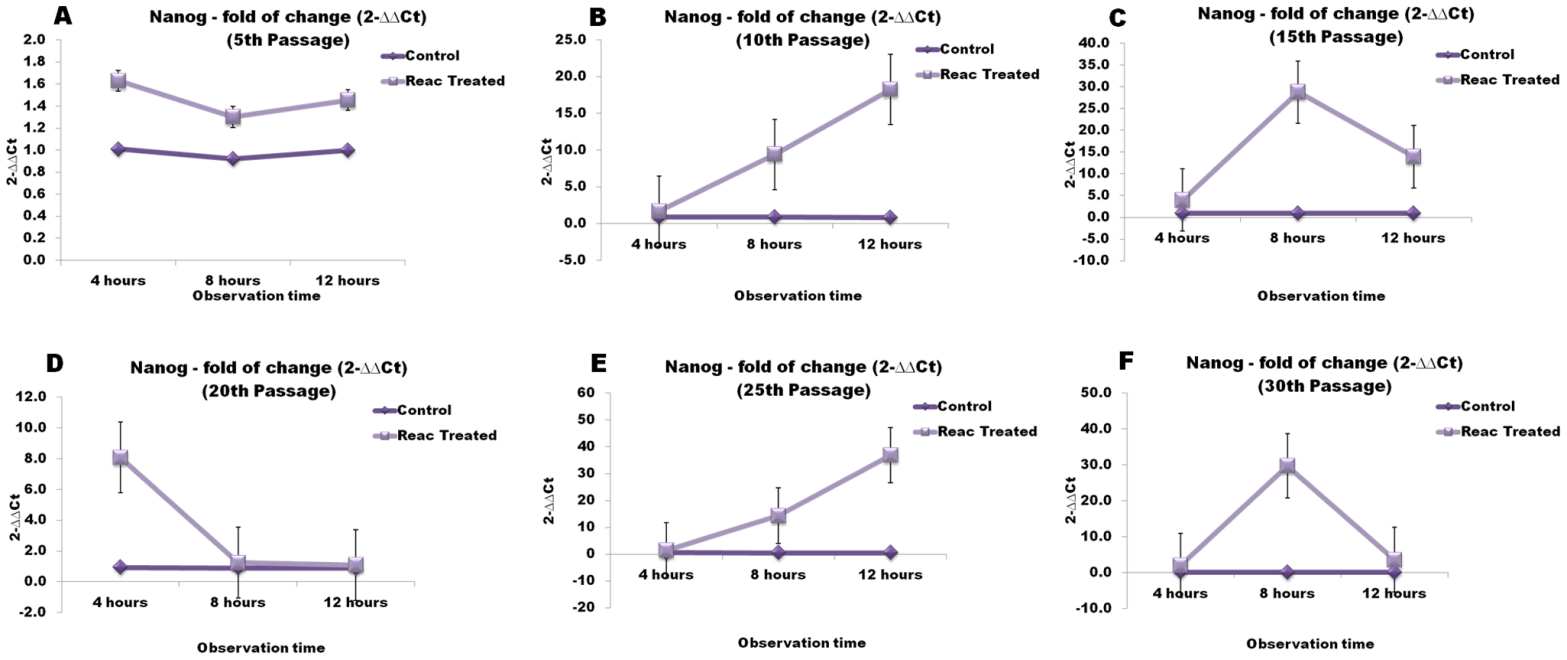
REAC TO-RGN treatment modulates the gene expression of Nanog. ADhMSCs at passages 5 (panel A), 10 (panel B), 15 (panel C), 20 (panel D), 25 (panel E) and 30 (panel F), reaching 80% confluence, were exposed for 4, 8 or 12 hours in the absence or presence of REAC. The amount of Nanog mRNA from control or REAC-treated cells was normalized to HPRT1, and was plotted as fold change relative to the mRNA expression at time 0, defined as 1. All the REAC-treated cells at each time point were significantly different from each control untreated cells (mean ± S.E.; n = 6) (P < 0.05).

**Figure 6:**
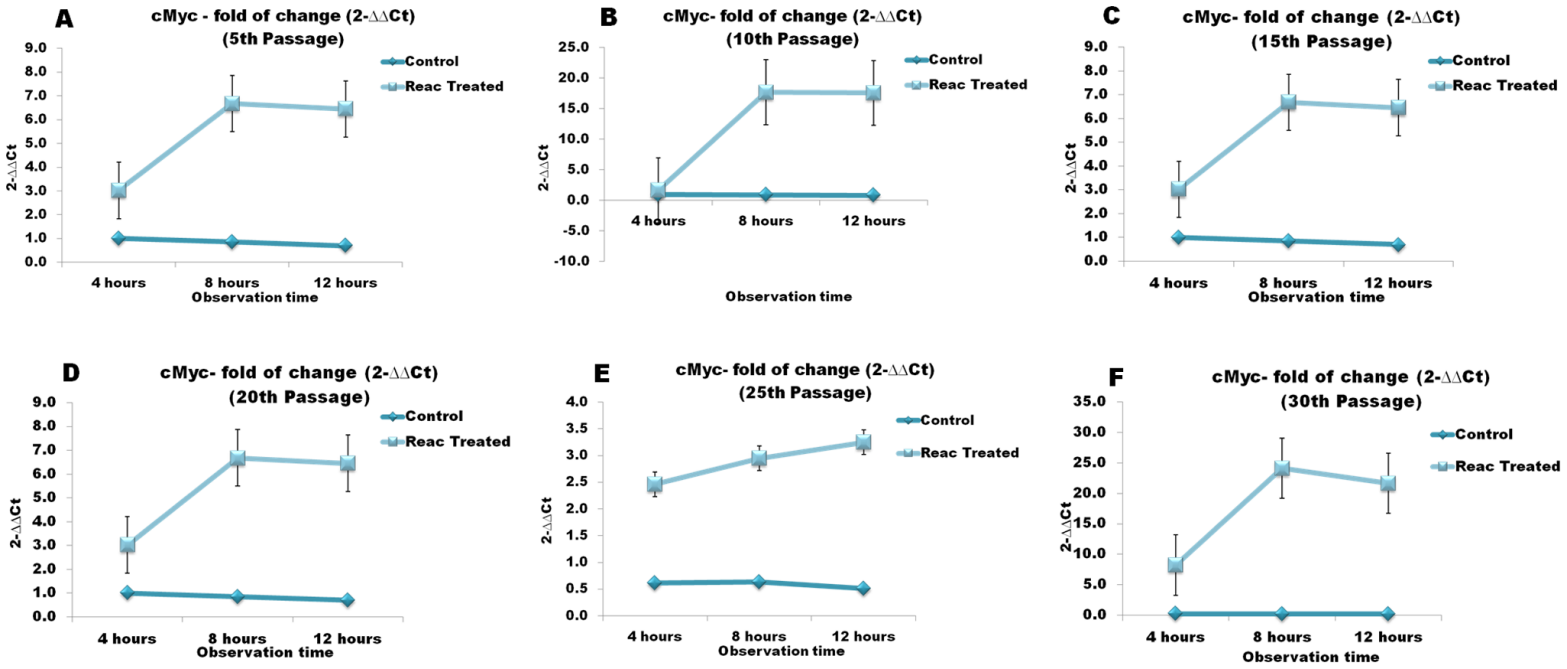
REAC TO-RGN treatment modulates the gene expression of cMyc. ADhMSCs at passages 5 (panel A), 10 (panel B), 15 (panel C), 20 (panel D), 25 (panel E) and 30 (panel F), reaching 80% confluence, were exposed for 4, 8 or 12 hours in the absence or presence of REAC. The amount of cMyc mRNA from control or REAC-treated cells was normalized to HPRT1, and was plotted as fold change relative to the mRNA expression at time 0, defined as 1. All the REAC-treated cells at each time point were significantly different from each control untreated cells (mean ± S.E.; n = 6) (P < 0.05).

**Figure 7:**
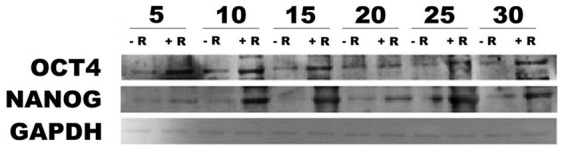
Effect of REAC TO-RGN treatment on the protein expression of Oct4 and Sox2. Total lysates were isolated from ADhMSCs, exposed for 12 hours in the absence (-R) or presence of REAC (+R), at passages 5, 10, 15, 20, 25, and 30. Samples were analyzed by Western blot, using polyclonal antisera against Oct4, Sox2 and GAPDH. The sizes of the bands were determined using prestained marker proteins. The data presented are representative of five separate experiments.

Over-passage transcriptional analysis shown that REAC TO-RGN exposure was able to finely tune the transcription of master stemness regulators. In particular, during a 4- to 12-hour exposure period, the gene expression of Oct4, Sox2, Nanog, and cMyc was significantly increased in REAC-exposed ADhMSCs at passages 10, 15, 20, 25 and 30, as compared to unexposed cells (Figures 3,4,5,6). As shown in Figure 7, Western blotting analysis revealed that REAC treatment also promoted Oct4 and Sox2 protein expression.

### REAC TO-RGN directly influences the expression of TERT and Telomere length during aging of ADhMSCs

**Figure 8:**
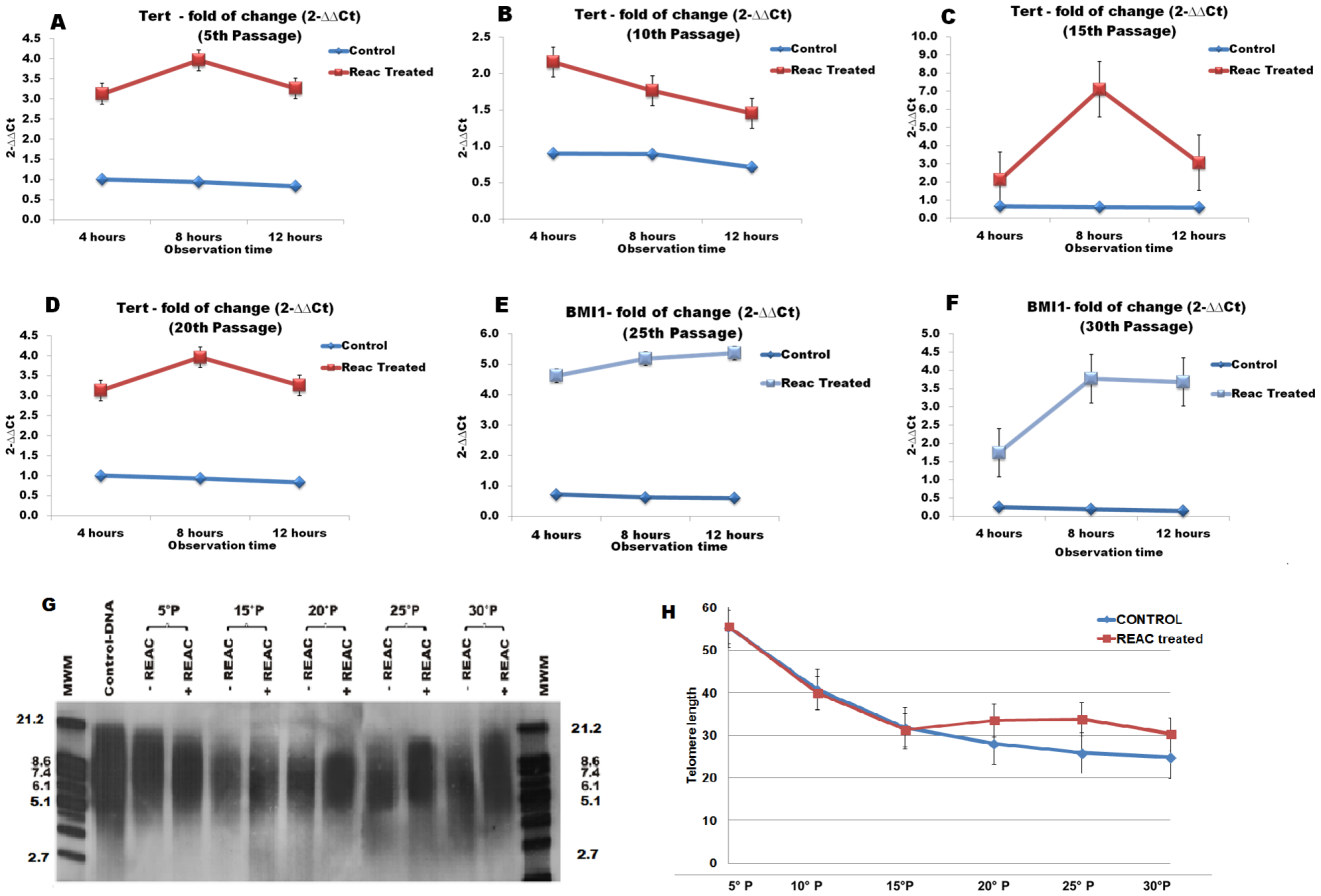
Effect of REAC *TO-RGN* exposure on TERT gene expression and telomere length. ADhMSCs at passages 5 (panel A), 10 (panel B), 15 (panel C), 20 (panel D), 25 (panel E) and 30 (panel F), reaching 80% confluence, were exposed for 4, 8 or 12 hours in the absence or presence of REAC. The amount of TERT mRNA from control or REAC-treated cells was normalized to HPRT1, and was plotted as fold change relative to the mRNA expression at time 0, defined as 1. All the REAC-treated cells at each time point were significantly different from each control untreated cells (mean ± S.E.; n = 6) (P < 0.05). To assess telomere length (panels G, H), cells at passages 5 (lanes 3, 4), 15 (lanes 5, 6), 20 (lanes 7, 8), 25 (lanes 9, 10), and 30 (lanes 11, 12), reaching 80% confluence, were exposed for 12 hours in the absence or presence of REAC. Lanes 1 and 13, molecular weight markers (MWM). Lane 2, telomere length assessed at basal conditions (defined as ADhMSCs cultured at passage 1). G, mean telomere length, assessed as described by telomere length assay (representative of 6 individual experiments). H, calculated values of mean telomere length (TRF was determined as described in the Methods section) (mean ± S.E.; n = 6). *significantly different from untreated controls (P < 0.05).

The gene expression of the catalytic subunit of telomerase (TERT) was assessed by real-time PCR in ADhMSCs cultured at passages 5, 10, 15, 20, 25, and 30 in the absence or presence of REAC treatment that was applied for 4, 8 or 12 hours. TERT expression decreased throughout the investigated culturing passages in control ADhMSCs, while it was remarkably retained at high levels in REAC TO-RGN exposed cells at all the investigated passages (Figure 8 panels A–F). Such transcriptional response was not significantly affected by the exposure period at early passages, when a 4-hour treatment was sufficient to achieve a maximal increase in TERT mRNA levels (Figure 8 panels A, B). At late passages, including passages 25 and 30, a more prolonged, 8–12-hour exposure was required to achieve the highest enhancement in TERT transcription (Figure 8 panels C–F).

Figure 8 also shows the analysis of telomere length during different passages in culture (panels G, H). It is evident that control, untreated ADhMSCs underwent a progressive reduction in telomere length along the different passages, as compared with ADhMSCs cultured at passage 1 (defined as “basal conditions”). Telomere length declined in both untreated and REAC exposed cells between passages 5 and 20. However, a significant, although incomplete, length recovery could be observed at the late 20, 25 and 30 passages only in REAC TO-RGN exposed (12 hours) ADhMSCs, when compared to basal conditions (Figure 8 panels G, H).

## Discussion

Several studies have provided evidence that the ability of stem cells to respond to environmental demands may be diminished during aging, suggesting that abnormalities in the regulation of stem cell homeostasis (proliferation, differentiation and survival) may contribute to aging and age-related diseases^37^,^38^,^39^,^40^. In terms of aging modulation, stem cells appear to play an important role virtually within all tissues, even those, such as the cerebral cortex and the heart, in which the cellular turnover in adults is exceedingly low^41^. In the last few years, there has been an increasing effort in exploiting the therapeutic potential of stem cells for the treatment of irreversibly damaged tissues that could not be rescued even by the most advanced pharmacological or surgical treatments. Despite the fact that hMSCs can be harvested from an increasing number of tissue sources, they are particularly scarce in the body. Analysis of currently available cell therapy protocols reveals that hMSCs are transplanted at high doses usually ranging between 10 and 400 million hMSCs per treatment (www.clinicaltrials.gov). As a consequence, these cells need to be expanded *in vitro* for multiple passages and prolonged time in culture – 8–12 weeks – before implantation. Within this context, the development of innovative approaches capable of counteracting several major traits of hMSC senescence during their expansion *in vitro* may be of particular relevance to the clinical translation of cell therapy strategies. ADhMSCs, due to the minimal invasiveness of fat harvesting and their multilineage potential are increasingly transferred into clinical trials.

In the current study, we show that exposure of ADhMSCs to an innovative device conveying radio electric fields is able to decrease the number of cells positively stained for the senescence marker SA-β-gal, and afford a significant rescue of two major mechanisms counteracting cell senescence during prolonged passages in culture. The REAC TO-RGN action did not require cumbersome gene manipulation through viral vector mediated gene transfer, or expensive synthetic chemistry. In particular, the ability of REAC TO-RGN to efficiently antagonize the down-regulation of Bmi1 transcription during multiple ADhMSC passages *in vitro* suggests the possibility to use a physical milieu to control a part of the molecular patterning that regulates cell senescence throughout chromatin remodeling processes. The relevance of such a reversal is highlighted by the observation that Bmi1 is transcriptionally down-regulated when cells undergo replicative senescence^6^,^16^,^42^. We found that REAC TO-RGN exposure increased the transcription of Oct4, Sox2, Nanog, and cMyc, the latter being also an important positive regulator of the Bmi1 gene^17^, and enhanced the expression of stemness related proteins, including Oct4 and Sox2. These observations indicate that the REAC technology can actively interfere with a complex molecular circuitry responsible for stem cell self-renewal, involving both telomerase-independent regulation of cellular senescence, and cell pluripotency.

These results are consistent with our previous observations showing that REAC TO-RGN exposure modulated the expression of Nanog, Sox2, Oct4, and cMyc in mouse embryonic stem cells, as well as in human skin-derived fibroblasts^31^,^32^. Furthermore, REAC TO-RGN exposure proved effective in antagonizing the aging process *in vitro*^33^,^34^,^35^. Here, we show that REAC TO-RGN exposure also affected telomerase-dependent senescence mechanisms, by transcriptionally enhancing the expression of TERT, and counteracting telomere shortening. Studies on brain development in mice have correlated a decrease in TERT expression and activity with decreased neuroblast proliferation, and differentiation^43^. Moreover, it has been demonstrated that MSCs or bone marrow stromal stem cells lacking telomerase activity undergo premature cellular senescence, with a progressive decline in the expression of early mesenchymal stem cell markers^44^. In this study, we observed that while during the first passages the REAC-mediated increase in TERT transcription did not occur as a function of time exposure, at late passages (20–30) a maximal transcriptional response resulted as a consequence of the REAC TO-RGN exposure-time. This is probably due to the accumulation of time required for counteracting the decline in the expression of antiaging genes that becomes more and more accentuated with over-passage culturing. Of note, the REAC TO-RGN treatment, while not exerting an appreciable effect on the telomere shortening that spontaneously occurs at early passages, was conversely able to oppose the telomere shortening that develops at late passages, when cell senescence in culture becomes more pronounced. Studies are on the way to further dissect the molecular mechanisms underlying the effect of REAC TO-RGN exposure on cell senescence *in vitro*, and to assess the REAC TO-RGN action in animal models of geriatric disorders *in vivo*.

## Methods

### Ethics statement

According to the policy approved by the local ethical committee of the University of Bologna (Title of approved project: Assessment of mesenchymal stem cells in human adipose tissue, code number 013/2010/O/Tess, date of approval from Ethical Committee: 16/02/2010), all tissue samples were obtained after informed consent. All participants, according to the consent procedure approved by the Ethical Committee, provided their written consent specifically dedicated to the harvesting of their abdominal fat (50–100 ml of lipoaspirate).

### Description of Radio Electric Asymmetric Conveyer (REAC) technology

The REAC is an innovative-patented technology^29^,^30^ for bio-stimulation and/or bio-enhancement techniques. The REAC device generates an emission of microwaves of very weak intensity. REAC technology is effective at very low power levels, approximately 2 mW at the emitter. Higher powers alter its mechanism of action. REAC technology is independent of the radiofrequency emission used. For REAC devices only the frequencies 2.4 and 5.8 GHz are used, because they are the most widespread and authorized at international level (i.e. Wi-Fi). The peculiarity of REAC technology is not the emission, but the particular physic link between the device and the cell culture or patient's body. This link is represented by the probe-asymmetric conveyer. Solely thanks to the conveyer (asymmetric probe) this emission can interact with the biological tissues of the human body, without depth limits and in a very innovative way. This interaction generates in tissues induced radiofrequency micro currents, variables according to the molecular characteristics of the tissues. The sum of these induced radiofrequency micro currents gives rise to a resulting micro current, generated by the body of the subject treated. This resulting micro current, concentrated by the probe-asymmetric conveyer of the device, exerts a therapeutic effect.

When the REAC apparatus was set at a frequency of 2.4 GHz and its conveyer asymmetric electrodes were immersed into the culture medium, the distance between the emitter at 2.4 GHz and the culture medium was approximately 35 cm. The electromagnetic quantities have been measured with the spectrum analyzer Tektronix model 2754 p, orienting the receiving antenna for maximum signal. With duration of single radiofrequency burst of 250 ms we have obtained the following results: Radiated power is about 2 mW, Electric field E = 0.4 V/m, Magnetic field 1 mA/m, Specific Absorption Rate - SAR 0.128 μW/g; determinate σ = 1 A/Vm and ρ = 1000 Kg/m3 the density of radio electric current flowing in the culture medium during the REAC single radiofrequency burst is **J** = 30 μA/cm2. The model used in this study (B.E.N.E Bio enhancer - Neuro enhancer, ASMED, Florence, Italy) is specific for regenerative treatments.

### REAC tissue optimization - regenerative treatments (TO-RGN)

REAC tissue optimization (TO) regenerative treatments (RGN) includes a set of treatment protocols, which are carried out by covering the treated area with a laminar electrode. In the case of cell cultures the laminar electrode is immersed in the medium. The REAC TO-RGN, used in this study, consists of a sequence of 250 milliseconds radiofrequency bursts.

### Isolation and culture of hADMSCs

According to the policy approved by the local Ethical Committee, all tissue samples were obtained after informed consent. Human subcutaneous adipose tissue samples were obtained from lipoaspiration/liposuction procedures by 50-year-old female donors (n = 14). Their mean BMI was 25.47 and their mean age was 49.57. After washing, lipoaspirates were digested with 0.2% collagenase A type I solution (Sigma-Aldrich), under gentle agitation for 45 min at 37°C, and centrifuged at 2000 rpm for 10 min to separate the stromal vascular fraction (SVF) from adipocytes. If necessary, this fraction was treated with red blood cell lysis buffer for 5 min at 37°C, then centrifuged again. The supernatant was discarded, and the cell pellet was resuspended and seeded in culture flasks in DMEM-low glucose (Lonza) supplemented with 20% heat inactivated FBS, 1% penicillin-streptomycin, 2 mM L-glutamine, and incubated at 37°C in a humidified atmosphere with 5% CO_2_. When the cultures were near confluence, the cells were detached by treatment with trypsin, and seeded in six-well tissue culture plates at the appropriate passages. The REAC apparatus was placed into a CO_2_ incubator, was set at 2.4 GHz and its conveyer electrodes were immersed for 4, 8 or 12 hours into the culture medium of ADhMSCs at passages 5, 10, 15, 20, 25 and 30.

### Characterization of ADhMSCs by Flow Cytometry Analysis

ADhMSCs were harvested by treatment with 0.08% trypsin-EDTA and incubated with 1 μg/10^6^cells FITC-conjugated antibodies for 40 min at 4°C in the dark. The antibodies used were: SH2, SH3, SH4, anti-CD166, anti-CD14, anti-CD34, anti-CD44, and anti-CD45. After washing, cells were analyzed on a flow cytometer (FACSCalibur, Becton Dickinson, San Jose, CA, USA) by collecting 10,000 events and the data analyzed using the Cell Quest Software (Becton Dickinson). ADhMSCs were positively stained with SH2, which identifies an epitope of endoglin (CD105), and were recognized by the SH3 and SH4 antibodies, which identify epitopes on culture-expanded stromal cells and bind CD73, a molecule involved in B-cell activation. ADhMSCs were also positive for CD29, the beta-subunit of an integrin family behaving as the major receptor for extracellular matrix molecules, and for CD166, a hMSC marker not found in hematopoietic precursors, and were uniformly positive for the CD44 hyaluronate receptor. Conversely, antigen profiles were negative for the hematopoietic markers CD14 and CD34 and the leukocyte common antigen CD45.

### SA-β-Gal staining

SA-β-Gal staining was performed using a “Senescence-associated β-Galactosidase Staining Kit” (Cell Signaling). Briefly, ADhMSCs cultured at passages 5, 10, 15, 20, 25 and 30 were exposed for 12 hours in the absence or presence of REAC in 6-well plates (3 × 10^3^ cells per well). Subsequently, cells were fixed with fixative solution and then processed according to the manufacturer's instructions. The cells were then photographed under an inverted microscope at 100× magnification for qualitative detection of SA-βGal activity. The number of positive (blue) and negative cells was counted in five random fields under the microscope (at 200× magnification and bright field illumination), and the percentage of SA-βGal-positive cells was calculated as the number of positive cells divided by the total number of counted cells.

### Gene expression

Total RNA was isolated using Trizol reagent, according to the manufacturer's instruction (Life Technologies). Total RNA was dissolved in RNAase-free water and, for RT-PCR, cDNA was synthesized in a 50-μl reaction volume with 1 μg of total RNA and MuMLV reverse transcriptase (RT) according to the manufacturer's instruction (Life Technologies). Quantitative real-time PCR was performed using an iCycler Thermal Cycler (Bio-Rad). Two μl cDNA were amplified in 50-μl reactions using Platinum Supermix UDG (Life Technologies), 200 nM of each primer, 10 nM fluorescein (BioRad), and Sybr Green. After an initial denaturation step at 94°C for 10 min, temperature cycling was initiated. Each cycle consisted of 94°C for 15 s, 55–59°C for 30 s and 60°C for 30 s, the fluorescence being read at the end of this step. To evaluate the quality of product of real-time PCR assays, melting curve analysis was performed after each assay. Relative expression was determined using the “delta-CT method” with hypoxanthine phosphoribosyltransferase 1 (HPRT1) as a reference gene. The mRNA levels of control and REAC-exposed ADhMSCs were expressed as fold of change (2^−ΔΔCt^), relative to the mRNA levels of the enzyme evaluated in ADhMSCs at passage 5 when cells reached 80% confluence before starting the REAC TO-RGN treatment (time 0).

All primers used in this study were from Invitrogen and previously described^27^,^31^,^32^,^45^,^46^,^47^,^48^,^49^,^50^.

### Immunoblotting analysis

Cells cultured at passages 5, 10, 15, 20, 25 and 30 were exposed for 12 hours in the absence or presence of REAC in 6-well plates (3 × 10^3^ cells per well).

Total cell lysates from ADhMSCs were electrophoresed on 10% Novex Tris-glycine polyacrylamide gels (Invitrogen, CA), in MOPS SDS Running Buffer, using an XCell SureLockTM Mini-Cell, according to the instruction provided by the manufacturer. After protein transfer to nitrocellulose membranes (Life Technologies), membrane saturation and washing, the immunoreaction was carried out for 1 hour at room temperature in the presence of the primary antibody, antisera against Oct4 (Santa Cruz), Sox2 (Sigma) and GAPDH (Santa Cruz) diluted 1:1000. After additional washing, membranes were incubated with anti-rabbit horseradish peroxidase (HRP) conjugated secondary antibody (PIERCE). Targeted protein expression was assessed by a chemioluminescence detection system (ECL Western blotting detection reagents were from Amersham Biosciences).

### Assessment of telomere length

Genomic DNA was extracted, using Qiamp DNA blood mini kit (Qiagen), at passages 5, 15, 20, 25 and 30 from control cells and ADhMSCs exposed to REAC for 4, 8 and 12 hours. Telomere length was determined using the Telo TAGGG telomere length assay kit^51^ (Roche), according to the manufacturer's instructions. Briefly, genomic DNA was digested with a mixture of frequently cutting restriction enzymes, digested DNA fragments were then separated by gel electrophoresis and transferred to a nylon membrane by Southern blotting. The blotted DNA fragments were hybridized to a Dygoxigenin (DIG)-labeled probe, specific for telomeric repeats and incubated with a DIG-specific antibody covalently coupled to alkaline phosphatase. Finally the alkaline phosphatase on the antibody metabolizes CDP-star, a highly sensitive chemioluminescent substrate: this produces a visible signal that indicates the location of immobilized telomere probe (and hence the terminal restriction fragment (TRF) on the blot). The average TRF length has been determined by comparing the location of the TRF on the blot relative to a molecular weight standard. Chemioluminescent signal has been acquired using a BioRad VersaDoc Imaging System. Mean TRF length has been defined according to the following formula: 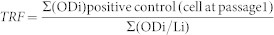


Where ODi is the chemioluminescent signal and Li is the length of TRF at position i.

### Data analysis

The statistical analysis of the data was performed by using the Statistical Package for Social Science (SPSS), version 13. For this study, were used nonparametric statistical tests: Friedman and Wilcoxon Signed Rank test. The first was used to detect differences in treatments across multiple test attempts, the second was used to evaluate, in the same group, the differences (Delta CT) between the data collected over a period of observation related to the treated and non-treated cells. Test, and all results P < 0.05 have been considered statistically significant.

## Author Contributions

S.R. and V.F. invented the REAC technology, and collaborated in conceiving the experimental plan. M.M. conceived and executed the experimental plan and wrote the manuscript. S.S., G.P., V.B. and A.C. performed the experiments. C.V. designed/supervised the project, and wrote the manuscript.
